# Supplementary material for: FcRn-Driven Nanoengineered Mucosal Vaccine with Multi-Epitope Fusion Induces Robust Dual Immunity and Long-Term Protection Against Brucella
Source: Vaccines (Basel). 2025 May 26;13(6):567. doi: 10.3390/vaccines13060567 (PMC12197398; doi:10.3390/vaccines13060567)
Supplement: Supplementary file 1 [file vaccines-13-00567-s001.zip › vaccines-3588679-supplementary.pdf]

Supplementary Materials-S1

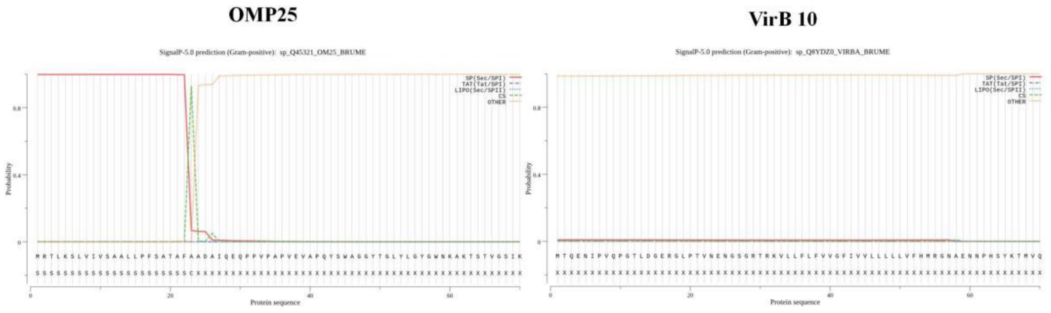

Figure S1: Signal peptide results of OMP25 and VirB 10 are presented.

Note: The Sec/SPI-type signal peptide cleavage site of Omp25 is located between amino acid residues 23 and 24 (AFA-AD), predicted with a confidence score of 0.9303, while no signal peptide domain was detected in VirB10.

Supplementary Materials-S2

The antigenicity, allergenicity, stability, hydrophilicity and toxicity of CTL, HTL, LBE and CBE epitopes

	Protein	Allele/Residues	Position	Epitopes	Score	Antigenicity	Allergenicity	Stability	Hydrophilicity	Toxicity
CTL	OMP25	HLA-A*03:01	56-65	AKTSTVGSIK	0.836911	0.7212	non-allergen	-9.98	-0.278	Non-Toxin
		HLA-A*11:01	57-66	KTSTVGSIKP	0.808395	0.7212	non-allergen	-9.98	-0.278	Non-Toxin
		HLA-A*03:01	163-171	AKLTDNILGR	0.596833	1.1659	non-allergen	69.68	-0.489	Non-Toxin
	VirB10	HLA-A*11:01	71-80	TSTVPMRTFK	0.967152	0.7935	non-allergen	10.54	-0.367	Non-Toxin
		HLA-A*03:01	72-81	STVPMRTFKL	0.85472	0.7935	non-allergen	10.54	-0.367	Non-Toxin
		HLA-A*02:01	79-88	FKLPPPPPPA	0.836702	1.1	non-allergen	39.26	-0.878	Non-Toxin
HTL	OMP25	HLA-DRB1*07:01	49-63	LGYGWNKAKTSTVGS	0.6023	0.7134	non-allergen	24.59	-0.527	Non-Toxin
		HLA-DRB1*07:01	50-64	GYGWNKAKTSTVGSI	0.596	0.5134	non-allergen	24.59	-0.48	Non-Toxin
	VirB10	HLA-DRB1*15:01	306-320	IETLGRYATQKVGGG	0.9514	0.5572	non-allergen	39.49	-0.36	Non-Toxin
		HLA-DRB1*03:01	265-279	PNGVVIDLDSPGADP	0.7914	0.8696	non-allergen	30.13	-0.127	Non-Toxin
		HLA-DRB1*07:01	366-380	ARDLDFSSVYDVKPK	0.5829	1.1873	non-allergen	20.96	-0.7	Non-Toxin
LBE	OMP25	/	45-60	TGLYLGYGWNKAKTST	0.91	0.2621	non-allergen	23.68	-0.594	Non-Toxin
			165-180	TDNILGRVEYRYTQYG	0.83	0.6037	non-allergen	13.56	-1.038	Non-Toxin
			182-197	KNYDLAGTTVRNKLDT	0.82	1.0977	non-allergen	26.68	-1.031	Non-Toxin
			235-250	ERGSTISGEYDANVKQ	0.92	1.3233	non-allergen	27.16	-1.238	Non-Toxin
	VirB10		175-190	HGTQTDARMASLLRNR	0.9	0.9742	non-allergen	20.34	-1.044	Non-Toxin
			309-324	LGRYATQKVGGGGSNQ	0.87	1.4924	non-allergen	22.56	-0.869	Non-Toxin
			83-98	PPPPPAPPEPPAPPPA	0.86	0.4204	non-allergen	38.76	-1.081	Non-Toxin
			280-295	LGGAGLPGYIDSHFWK	0.84	0.5534	non-allergen	24.07	-0.006	Non-Toxin
CBE	VirB10		122-239	A:E123, A:S124, A:T127, A:	0.734	0.5205	non-allergen	38.57	-0.13	Non-Toxin

	F128, A:G131, A:G132, A:R145, A:G146, A:K147, A:D148, A:G154, A:L163, A:S166, A:H167, A:I168, A:A169, A:K170, A:S171, A:H176, A:R177, A:R178, A:D179, A:G180, A:F181, A:R182, A:A183, A:E184, A:K185, A:I186, A:M187, A:Q188, A:D189, A:R190, A:L191, A:L192, A:S193, A:R194, A:E195, A:N196, A:V197, A:S198, A:V199, A:V200, A:W201, A:N202, A:S203, A:V204, A:D206, A:E207, A:L209, A:G210, A:T211, A:E212, A:A213, A:R214, A:G218, A:A219, A:T220, A:V221, A:T222, A:G223, A:V224, A:R225, A:L226, A:K227, A:N228, A:I229, A:V230, A:T231, A:G232, A:E233, A:T234, A:Q235, A:E236, A:R237, A:A238, A:T239, A:H240	

Note: Analysis summary of all selected epitopes after screening, including CTL, HTL, LBE and CBE.

Supplementary Materials-S3

Before mutation

DKTHTCPPCPAPELLGGPSVFLFPPKPKDTLMISRTPEVTCVVVDVSHEDPEVKFNW
YVDGVEVHNAKTKPREEQYNSTYRVVSVLTVLHQDWLNGKEYKCKVSNKALPAPIE
KTISKAKGQPREPQVYTLPPSRDELTKNQVSLTCLVKGFYPSDIAVEWESNGQPENNY
KTTPPVLDSDGSFFLYSKLTVDKSRWQQGNVFSCSVMHEALHNHYTQKSLSLSPGK

After mutation

DKTHTSPPSPAPELLGGPSVFLFPPKPKDTLYITREPEVTCVVVDVSHEDPEVKFNW
YVDGVEVHNAKTKPREEQYNSTYRVVSVLTVLHQDWLNGKYCVSNKALPAPIEKT
ISKAKGQPREPQVYTLPPSRDELTKNQVSLTCLVKGFYPSDIAVEWESNGQPENNYK
TTPPVLDSDGSFFLYSKLTVDKSRWQQGNVFSCSVMHEALKFHYTQKSLSLSPGK

Figure S2. Engineering of human IgG1 Fc-derived h-tFc through site-directed mutagenesis.

(A) Human IgG1 Fc domain with critical functional regions highlighted.

(B) Modified h-tFc with three key mutagenesis strategies: Blue: C226S/C229S substitutions disrupting interchain disulfide bonds to enforce monomeric conformation. Red: Deletion of E318A/K320A/K322A will break the C1q binding motif (aa 318 - 322), eliminating complement activation in humans and mice. Green: Efgartigimod-inspired modifications (M252Y/S254T/T256E/H433K/N434F) enhancing pH-dependent FcRn binding affinity while reducing effector functions.

Supplementary Materials-S4

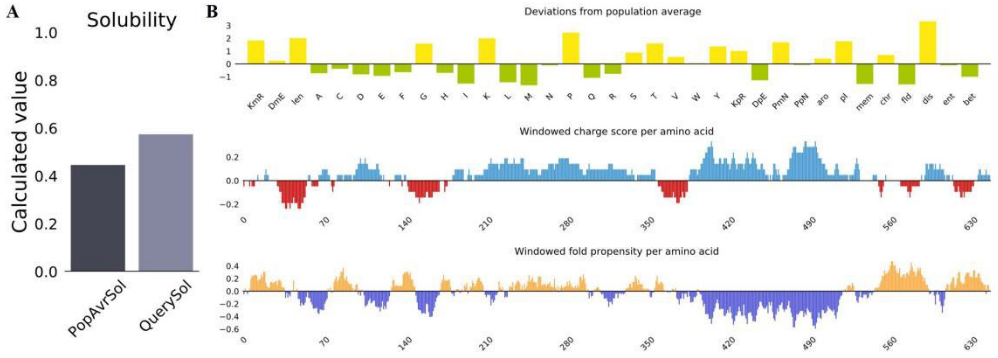

Figure S3. Validation of sequence-based solubility prediction model

(A) "Scatter plot comparing experimental solubility (%) versus computational predictions (%). The red dashed line indicates the optimal classification threshold at 58% solubility, with the gray band representing the population average benchmark (PopAvrSol = 0.45). Data points include three independent datasets: Escherichia coli proteome (n=679), crystallization propensity test set (n=200), and UniProt-derived sequences (n=1,294), showing a Pearson correlation coefficient (r) of 0.621. Color gradient corresponds to QuerySol normalized values, where scores >0.45 (warmer colors) indicate above-average solubility relative to experimental observations."

(B) Surface physicochemical property mapping

"Electrostatic potential and hydrophobicity distribution visualized on protein structure (PDB ID: [insert]) using protein-sol patches algorithm. Blue regions: positive charge clusters (>+0.5 $kT \cdot e^{-1}$), red areas: negative charge concentrations (<-0.5 $kT \cdot e^{-1}$), yellow patches: hydrophobic domains (Wimley-White interfacial scale >3.0). Color scales were dynamically normalized based on the input structure's calculated electrostatic potential range."

Supplementary Materials-S5

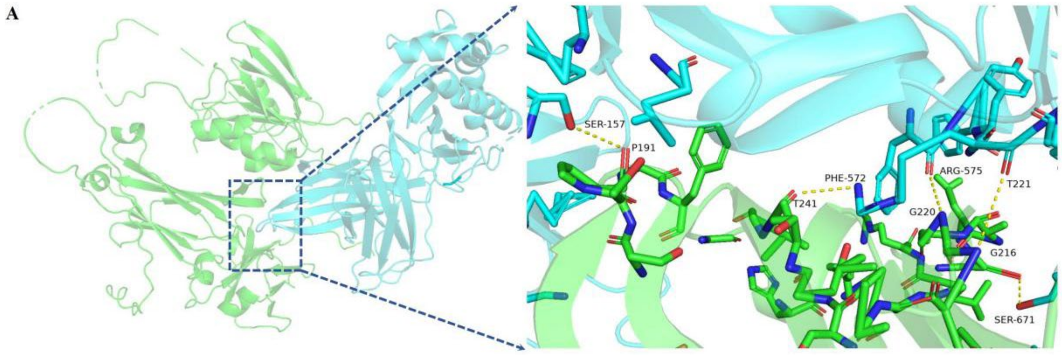

Figure S4-A. Molecular docking interface between h-tFc-MEV and FcRn.

Structural visualization (PyMOL v2.5.0) of the predicted binding interface generated by AlphaFold v2.3.2, highlighting complementary surface features and key interaction residues.

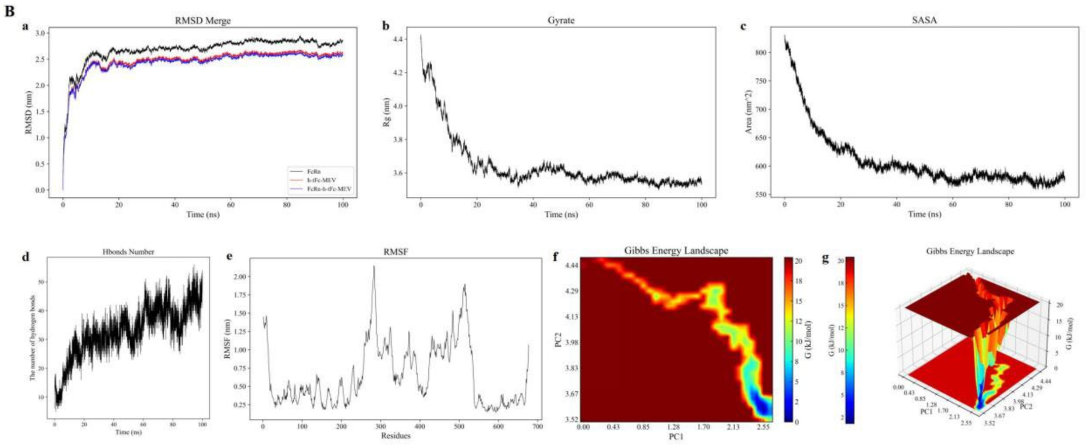

Figure S4-B. Molecular dynamics simulation.

(a) RMSD trajectories (0.1 – 0.3 nm post-equilibration at 15 ns). **(b)** Radius of gyration (Rg) reduction (17%). (c) Solvent-accessible surface area (SASA = 600 nm²). **(d)** Hydrogen bond persistence (28 – 33 bonds) at the binding interface. **(e)** RMSF profiles showing restricted fluctuations (<2.0 Å) at binding residues versus flexible loops. **(f) 2D.** Free energy basin distribution for h-tFc-MEV (concentrated) versus unmodified system (dispersed). **(g) 3D.** Free energy basin distribution for h-tFc-MEV (concentrated) versus unmodified system (dispersed).

Supplementary Materials-S6

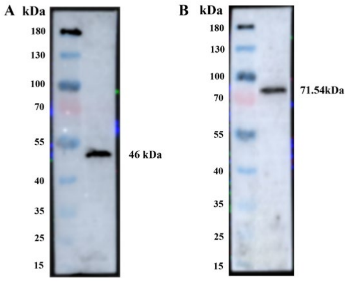

Figure S5. Heterologous expression of recombinant MEV and h-tFc-MEV protein in E. coli.

(A) Lane 1: Prestained protein ladder (kDa); Lane 2: Western blot analysis using anti-His tag antibody revealed a 46 kDa band (Lane 2) corresponding to the predicted molecular weight of MEV-His fusion protein. **(B)** Lane 1: Prestained protein ladder (kDa); Lane 2: Western blot analysis using anti-His tag antibody revealed a 71.54 kDa band corresponding to the predicted molecular weight of h-tFc-MEV-His fusion protein.

Supplementary Materials-S7

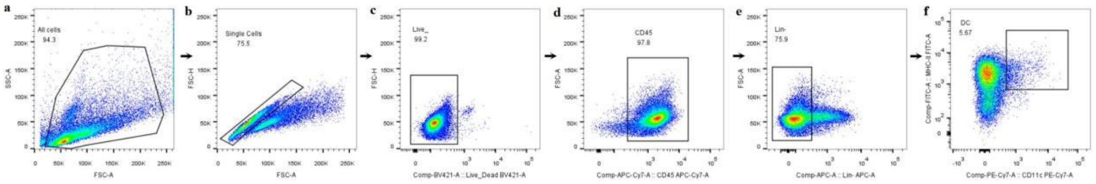

Figure S6. Gating strategy for dendritic cell (DC) subset analysis by flow cytometry:

(a) Debris exclusion and single-cell population gating based on forward scatter area (FSC-A) and side scatter area (SSC-A); **(b)** Doublet exclusion using FSC height (FSC-H) vs. FSC-A; **(c)** Viable cell selection via negative staining with live/dead dye (e.g., DAPI or 7-AAD); **(d)** Identification of $CD45^{+}$leukocyte population; **(e)** Exclusion of non-target immune cells (T cells, B cells, NK cells) by staining with LIN-APC: CD3, CD19, and CD49b antibodies; **(f)** Mature DC subsets defined as $CD11c^{+}MHC\text{-}II^{+}$

double-positive population (representative gating strategy shown).

Supplementary Materials-S8

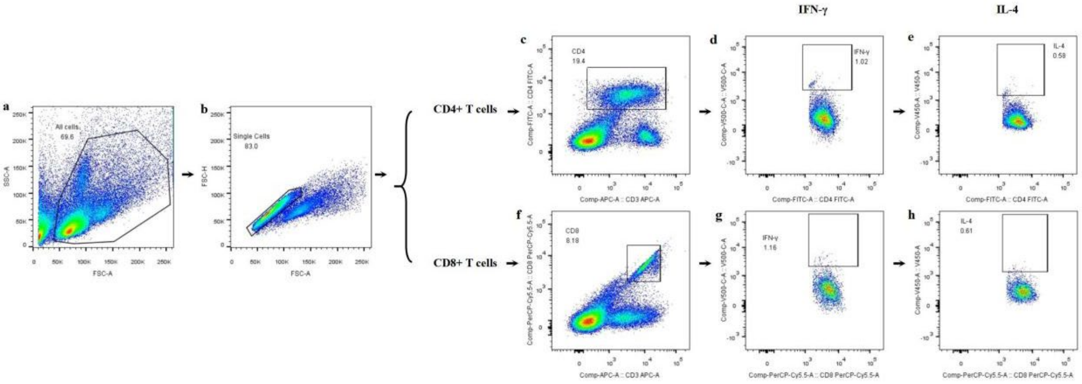

Figure S7. Gating strategy for analysis of IFN-γ⁺IL-4⁺ CD4⁺ and CD8⁺ T cells

(a) Lymphocytes were gated based on forward scatter (FSC-A) vs. side scatter (SSC-A) to exclude debris and granulocytes.

(b) Single-cell events were selected using FSC-height (FSC-H) vs. FSC-area (FSC-A) to eliminate doublets.

(c) CD3⁺ T cells were isolated from the lymphocyte gate, followed by subset differentiation of CD4⁺ T cells.

(d-e) Intracellular staining for IFN-γ and IL-4 was performed on CD4⁺ T cells.

(f) CD8⁺ T cell subsets were defined within CD3⁺ T cells.

(g-h) Intracellular detection of IFN-γ and IL-4 in CD8⁺ T cells.

Supplementary Materials-S9

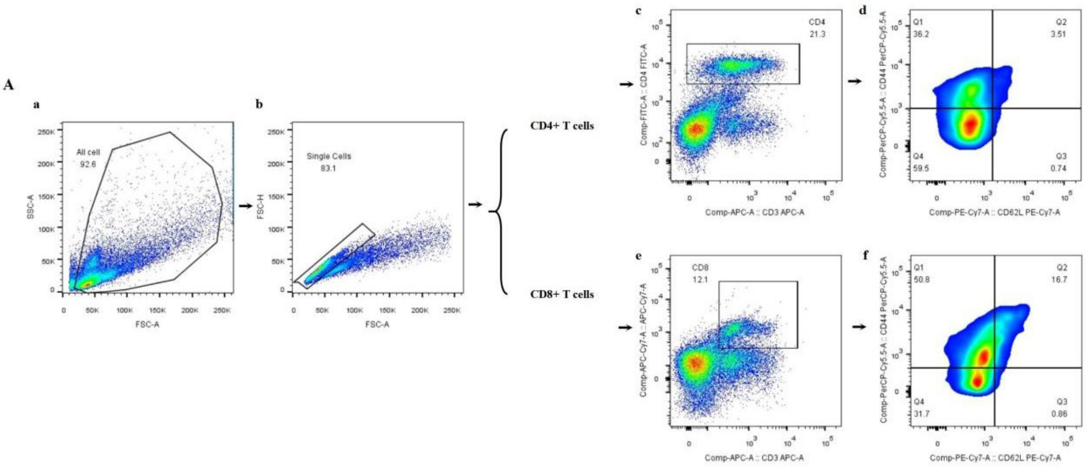

Figure S8-A. Gating strategy for analysis of CD4⁺/CD8⁺ T cell subsets and central memory T cells (TCM)

(a) Lymphocytes were gated based on forward scatter (FSC-A) vs. side scatter (SSC-A) to exclude debris and granulocytes.

(b) Single-cell events were selected using FSC-height (FSC-H) vs. FSC-area (FSC-A) to eliminate doublets.

(c) CD3⁺ T cells were gated from lymphocytes, followed by identification of CD4⁺ T cell subsets.

(d) CD4⁺ TCM cells (CD44⁺CD62L⁺) were analyzed within the CD4⁺ T cell population.

(e) CD8⁺ T cell subsets were defined within CD3⁺ T cells.

(f) CD8⁺ TCM cells (CD44⁺CD62L⁺) were identified from the CD8⁺ T cell population.

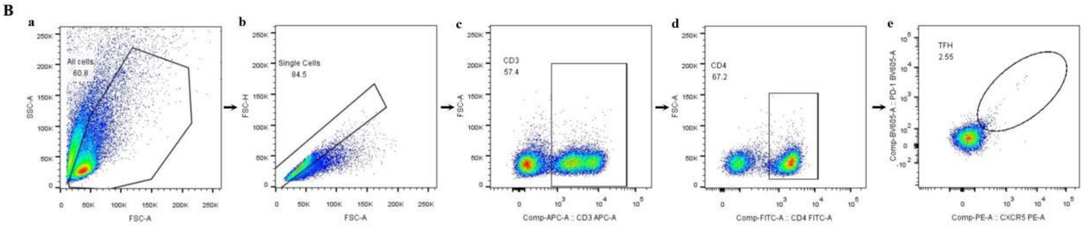

Figure S8-B. **Gating strategy for follicular helper T cell (TFH) analysis**

(a) Lymphocytes were gated based on forward scatter (FSC-A) vs. side scatter (SSC-A) to exclude debris and granulocytes.

(b) Single-cell events were selected using FSC-height (FSC-H) vs. FSC-area (FSC-A) to eliminate doublets.

(c) $CD3^+$ T cells were isolated from the lymphocyte gate.

(d) $CD4^+$ T cell subsets were further identified within the $CD3^+$ T cell population.

(e) TFH cells ($CXCR5^+PD\text{-}1^+$) were analyzed within the $CD4^+$ T cell population.

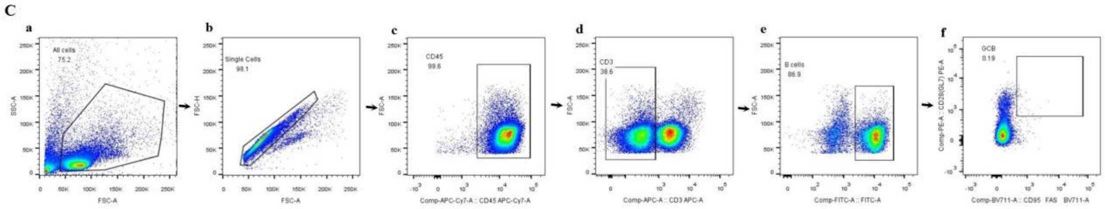

igure S8-C. **Gating strategy for germinal center B cell (GCB) analysis**

(a) Lymphocytes were gated based on forward scatter (FSC-A) vs. side scatter (SSC-A) to exclude debris and granulocytes.

(b) Single-cell events were selected using FSC-height (FSC-H) vs. FSC-area (FSC-A) to eliminate doublets.

(c) $CD3^-$ cell populations were isolated from the lymphocyte gate.

(d) B cell subsets ($B220^+$) were further identified within the $CD3^-$ population.

(e) Germinal center B cells ($GL7^+FAS^+$) were analyzed within the $B220^+$ B cell population.
